# Elevated empathy in adults following childhood trauma

**DOI:** 10.1371/journal.pone.0203886

**Published:** 2018-10-03

**Authors:** David M. Greenberg, Simon Baron-Cohen, Nora Rosenberg, Peter Fonagy, Peter J. Rentfrow

**Affiliations:** 1 The City College and Graduate Center, City University of New York, New York, New York, United States of America; 2 Autism Research Centre, Department of Psychiatry, University of Cambridge, Cambridge, United Kingdom; 3 Department of Psychology, University of Cambridge, Cambridge, United Kingdom; 4 Research Department of Clinical, Educational and Health Psychology, University College London, and The Anna Freud Centre, London, United Kingdom; Max-Planck-Institut fur Psychiatrie, GERMANY

## Abstract

Traumatic events increase the risk of depression, but there is also evidence that adversity can lead to posttraumatic growth, including increased compassion and prosocial behavior. To date there is no empirical research pinpointing childhood trauma to an increase in trait empathy in adulthood. Although somewhat counter-intuitive, this might be predicted if trauma not only increases fear of future threat but also renders the individual more sensitive to suffering in others. We explored this possible link using multiple studies, self-report measures, and non-clinical samples. Results across samples and measures showed that, on average, adults who reported experiencing a traumatic event in childhood had elevated empathy levels compared to adults who did not experience a traumatic event. Further, the severity of the trauma correlated positively with various components of empathy. These findings suggest that the experience of a childhood trauma increases a person’s ability to take the perspective of another and to understand their mental and emotional states, and that this impact is long-standing. Future research needs to test if this is seen on performance measures, and how these findings extend to clinical populations.

## Introduction

Psychological and physical trauma can have profound effects on development and well-being throughout the life-course. Because traumatic events in childhood occur at key psychosocial and biological stages of development, their impact can continue into adult life. People who experience a childhood trauma are at risk for a variety of negative health outcomes, but paradoxically there are opportunities for personal growth [1,2]. Although there is an abundance of research on the negative outcomes of trauma, emerging evidence suggests that experiencing adversity can also increase posttraumatic growth including compassion and prosocial behavior [3,4]. However, research has yet to pinpoint if traumatic events, particularly in childhood, link to trait empathy in adulthood. We address this gap in the literature by testing if trait empathy differs for adults who experienced a childhood trauma compared to those who did not. If empathy is indeed altered in adults with a childhood trauma, this would suggest that its impact on empathy is long-standing and transgressed throughout adulthood.

Trauma and maltreatment can lead to an array of negative outcomes for the victim. These include aggressive and violent behavior [5], and depressive and psychotic symptoms [6,7]. Trauma and maltreatment can also lead to a variety of psychiatric conditions including borderline personality disorder (BDP), bipolar disorders, and major depression [8–11]. Childhood trauma and major depression is associated with atypical neuroanatomy in women [12], likely a consequence of the trauma and depression, and there are also genetic associations with childhood trauma and negative outcomes, including aggressive behavior and BPD [13–15], likely part of the complex causal risk factors.

Though research mainly focuses on the negative outcomes of trauma (often within clinical populations), recovery and resilience after a trauma is also observed in the general population [16]. Resilience in the aftermath of a trauma can result from varied factors and pathways, including ‘self-enhancement’, coping styles, and personality traits [17–19]. Resilience after a trauma is also associated with differences in neurobiology [20]. Further, trauma provides an opportunity for growth and transformation. That is, a person can show positive psychological changes and personal improvements after the trauma as a result of learning gained through coping with the trauma [21–23].

Surprisingly, there has been little research into the relationship between trauma and empathy. Empathy is the ability to recognize another’s thoughts and feelings, and to respond to these with an appropriate emotion [24]. Cognitive empathy is synonymous with theory of mind or ‘mind-reading’, the ability to place oneself in another person’s shoes, to imagine their mental and emotional states, and predict their behavior on the basis of these mental states [25]. Affective empathy is the drive to respond to another person’s mental states with an appropriate emotion [24]. Sympathy is a special case of affective empathy that reflects a person’s response to the distress of another, which may lead to attempts to alleviate their pain or suffering [24].

There is reason to expect that empathy may play a role in the aftermath of a traumatic event. Trauma increases attention to emotion [26], environmental cues [27], and increases amygdala responsiveness (which involves emotional attentiveness) [28–31]. This increase in awareness could improve the ability to recognize, understand, and react appropriately to these states in others, in comparison to individuals who have not had a traumatic experience. Yet, although there has been some work on clinician’s empathy when treating trauma victims, there has been almost no empirical work on the impact of trauma on the empathy of the victim [32,33]. Recent research by Lim and DeSteno [3] suggests that the severity of past adversity can lead to increased compassion and that this link is mediated by empathy. Research has also shown that adversity can be linked to an increase in prosocial and altruistic behavior [4,34]. This prior theory and research provides a rationale for why heightened empathy may be observed after traumatic events. Hence, we hypothesized that empathy in adulthood would be observed to be elevated for individuals with a childhood trauma compared to those without a trauma. Given that empathy has multiple facets, we predicted that both cognitive empathy and affective empathy would be elevated: cognitive empathy (also referred to as “mentalizing”) is the ability to understand another’s thoughts and feelings, whereas affective empathy is the ability respond to another person’s mental state with an appropriate emotion [24].

### Aims

In the present study we tested if traumatic experiences that occur exclusively during childhood link to empathy levels in adulthood. To address this question we conducted two studies. In Study 1 we asked adults to report if they had a history of childhood trauma and to complete a measure of trait empathy, the Empathy Quotient (EQ) [24]. In Study 2 we asked an independent sample of adults to complete the same task using a different empathy measure, the Interpersonal Reactivity Index (IRI) [35]. In each study, we examined the self-reported empathy of adults (on both the domain and facet level) who reported they had experienced a childhood trauma, compared to adults who did not. We also examined how the severity of the trauma, and the age at which the trauma occurred, correlated with empathy levels.

## Study 1

### Method

#### Participants

N = 387 adult participants were recruited via Amazon’s Mechanical Turk [36,37]. Participants ranged in age from 19 to 65 with a mean of 34.84 (*SD* = 11.72). 117 participants were male (30.2%), 269 (69.5%) were female and in 1 (0.3%) gender was unspecified. N = 302 (78%) were White. Studies 1 and 2 received ethical approval from the Cambridge Psychology Research Ethics Committee of the University of Cambridge. Information about participants’ prior clinical histories or diagnoses (e.g. history of depression) was not asked for.

#### Measures

**Empathy.** All participants completed the 40-item Empathy Quotient (EQ) [24]. The original 60-item EQ contains 20 items that are filler, thus we used the 40-item version to reduce potential participant fatigue. The EQ is a self-report measure of both the cognitive and affective components of empathy. Participants are asked to indicate their degree of agreement or disagreement for each statement on a four point scale (*strongly disagree*, *slightly disagree*, *slightly agree*, or *strongly agree*). For positively poled items, two points are given for strong agreement and one point is given for slight agreement. For negatively poled items, two points are given for strong disagreement and one point is given for slight disagreement. There are three facets that are recoverable from the EQ: Cognitive Empathy, Affective Empathy, and Social Skills [38–40]. Each of the three facets consists of five items [40]. Cognitive Empathy includes: “I am good at predicting how someone will feel”; “I can tune into how someone else feels rapidly and intuitively”; and “I can easily work out what another person might want to talk about”; Affective Empathy includes: “I really enjoy caring for other people”; “Seeing people cry doesn’t really upset me” (reverse coded); and “I tend to get emotionally involved with a friend’s problems”, and Social Skills includes: “I do not tend to find social situations confusing”; “I find it hard to know what to do in a social situation” (reverse coded); and “I often find it difficult to judge if something is rude or polite” (reverse coded). The reliabilities for the EQ in this study were α = .88 for total EQ score; α = 83 for Cognitive Empathy; α = .66 for Affective Empathy; and α = .64 for Social Skills. In terms of validity, the EQ correlates with behavioral tasks of empathy including the Reading the Mind in the Eyes Test (RMET) [39].

**Childhood trauma.** All participants completed a modified version of the Childhood Traumatic Events Scale [41], which asks about specific traumatic events that occurred prior to and including the age of 17. Specifically, participants were asked to indicate yes, no, or rather not say for the experience of five traumatic events: (1) Prior to the age of 17, did you experience a death of a very close friend or family member?; (2) Prior to the age of 17, was there a major upheaval between your parents (such as divorce, separation)?; (3) Prior to the age of 17, did you have a traumatic sexual experience (e.g., being raped, molested, etc.)?; (4) Prior to the age of 17, were you the victim of violence (e.g., child abuse, being mugged or assaulted—other than sexual)?; and (5) Prior to the age of 17, did you experience any other major upheaval that you think may have shaped your life or personality significantly? For each of these questions, participants were asked to indicate their age at which the trauma occurred (using six answer choices that have been used in previous research [19]: 0–2; 3–5; 6–8; 9–11; 12–14; 15–17), and to indicate the severity of the trauma on a scale from 1 (*not at all traumatic*) to 7 (*extremely traumatic*). These are the same age brackets used in previous research: there is no developmental rationale for bracketing the age ranges other than to condense the number of answer choices.

#### Statistical analysis

There were initially N = 410 participants who completed the test battery, however 23 of these participants indicated “rather not say” for one of the childhood trauma questions and since we did not know whether or not they had experienced that trauma, we did not include them in the analysis. Of the N = 387 participants that remained, 309 indicated that they experienced one or more of the childhood traumas (including ‘other’) and were designated to the “trauma group. N = 78 participants indicated that they did not experience any childhood trauma and were designated to the “no trauma group”.

### Results

#### Trauma vs. no trauma groups

**Fig 1:**
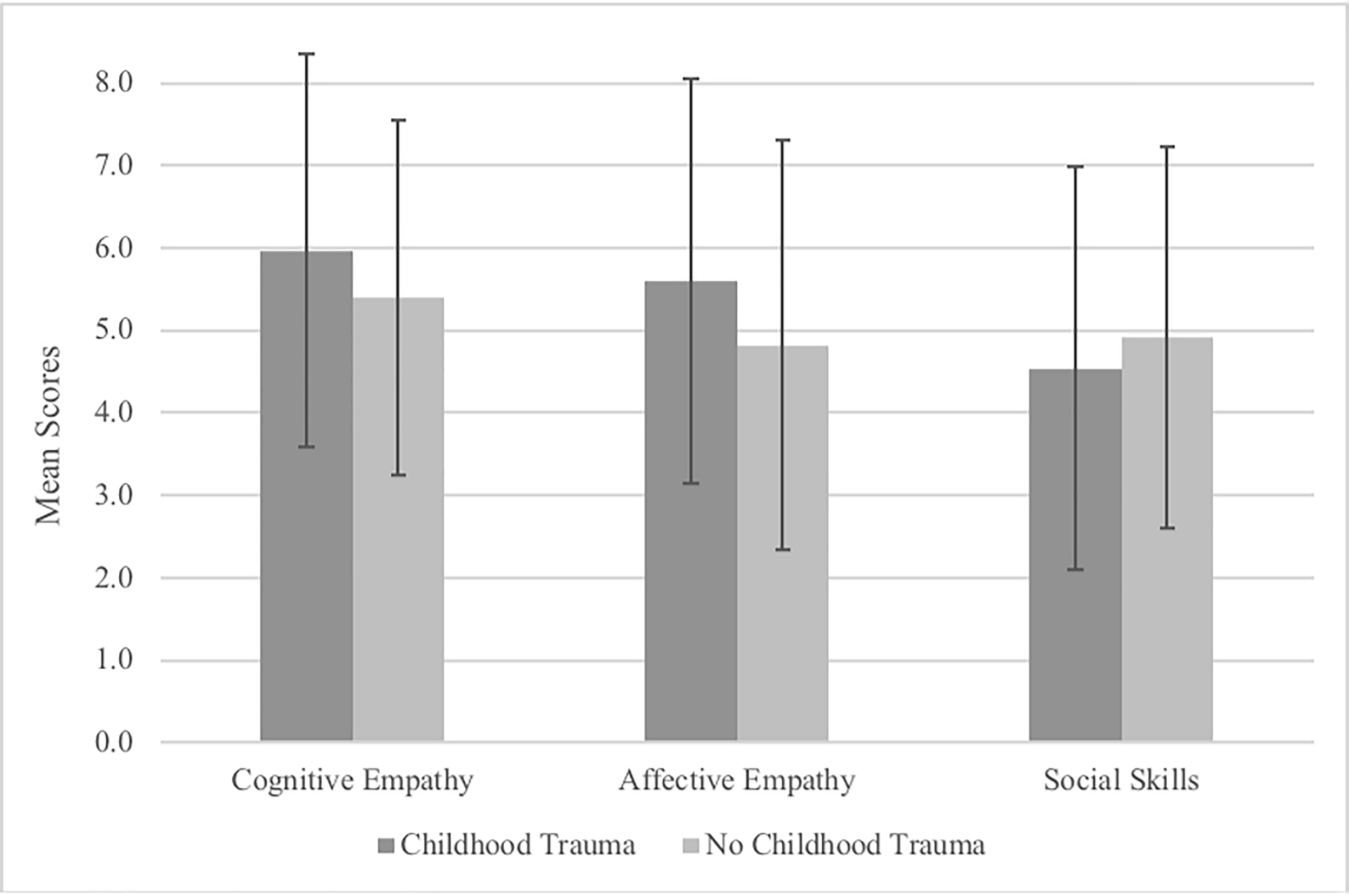
Differences in EQ facet scores by group type.

Previous research has shown evidence that there are sex differences in empathy with females, on average, scoring higher than males [42]. Therefore, we performed ACOVAs with group type as the IV, EQ scores as the DV, and sex as the covariate. There was a significant effect of group type on Affective Empathy (*F*(1, 385) = 5.48, *p* = .01; η_p_^2^ = .014), with the trauma group scoring higher than the no trauma group. There was no significant effect of group type on Cognitive Empathy (*F*(1, 385) = 3.20, *p* = .74, η_p_^2^ = .008), Social Skills (*F*(1, 385) = 1.67, *p* = .20, η_p_^2^ = .004), or total EQ scores (*F*(1, 385) = 1.47, *p* = .23). We tested the assumption of homogeneity of regression slopes by entering interaction terms into the models, which yielded nonsignificant results, and therefore the assumption was tenable. Together, these results suggest that the affective component of empathy is most strongly linked to empathy scores in adulthood. Mean EQ facets scores are presented in Fig 1 for each of the groups (error bars are based on *SD*).

#### Trauma severity

We next examined zero-order correlations between the severity of the specified traumas and EQ scores. As shown in Table 1, total EQ scores were significantly positively correlated with all trauma subgroups except for sexual abuse (*r* = .18, *p* = .11). Cognitive Empathy was positively correlated with the severity of the experience of the death of a family member or friend; Affective Empathy was positively correlated with the severity of parental upheaval, sexual abuse, and ‘other’ traumas (*r*s < .20); and Social Skills was positively associated with the severity of violence.

**Table 1:** Correlations between EQ scores and severity of childhood traumas.

	EQ total	Cognitive Empathy	Affective Empathy	Social Skills
**Death**	.19**	.15*	.13	.11
**Parental upheaval**	.20*	.10	.20**	.09
**Sexual abuse**	.18	.09	.26*	.08
**Violence**	.27*	.08	.10	.25*
**Other**	.24*	.10	.23*	.06

*Note*: Cell entries are zero-order correlations between severity of childhood traumas and self-rated empathy scores. Ns = 188 (Death); 163 (Parental upheaval); 86 (Sexual abuse); 69 (Violence); and 107 (Other).

*p < .05

** p < .01.

#### Age during the trauma

We next examined the links between empathy scores and the age at the time of the trauma. ANOVAs revealed that there were no significant effects of age during the trauma on empathy scores (*F*s(5, 181) ranged from .61 for EQ total score to 1.30 or Social Skills) and this result was further confirmed by post-hoc Tukey tests testing comparisons between each individual age group, with no significant differences between any of the groups.

## Study 2

The aim of this study was to replicate results from Study 1 using a separate sample and alternative empathy measure.

### Method

#### Participants and procedures

N = 442 adult participants were recruited via Amazon’s Mechanical Turk. Participants ranged in age from 18 to 65 with a mean of 34.94 (*SD* = 11.90). N = 162 (32%) participants were male, 277 were female (62%), and 3(1%) indicated unspecified. N = 350 (79%) indicated they were White. The procedures in Study 2 were the same as in Study 1.

#### Measures

**Empathy.** All participants completed the 28-item Interpersonal Reactivity Index (IRI) [35], which is a measure of dispositional empathy. The IRI consists of a total scale score labelled as Global Empathy, and four facet scales: Perspective Taking, Empathic Concern, Fantasy, and Personal Distress. Each facet consists of seven items. Perspective Taking is most closely reflective of the Cognitive Empathy facet of the EQ, and Empathic Concern is most closely reflective of the Affective Empathy facet of the EQ. The reliabilities for the IRI in this study were α = .85 for Global Empathy; α = 81 for Perspective Taking; α = .82 for Fantasy; α = .86 for Empathic Concern; and α = .84 for Personal Distress. In terms of validity, the IRI correlates with behavioral tasks of empathy including the Multifaceted Empathy Test (MET) [43].

**Childhood trauma.** Traumatic events during childhood were assessed using the same measures as in Study 1.

#### Statistical analysis

There were initially N = 491 participants who completed the test battery, however 57 of these participants indicated “rather not say” for one of the childhood trauma questions and since we did not know whether or not they had experienced that trauma, we did not include them in the analysis. Of the 442 participants that remained, 348 indicated that they experienced one or more of the childhood traumas (including ‘other’) and were designated to the “trauma group”. N = 94 participants indicated that they did not experience any childhood trauma and were designated to the “no trauma group”.

### Results

#### Trauma vs. no trauma groups

**Fig 2:**
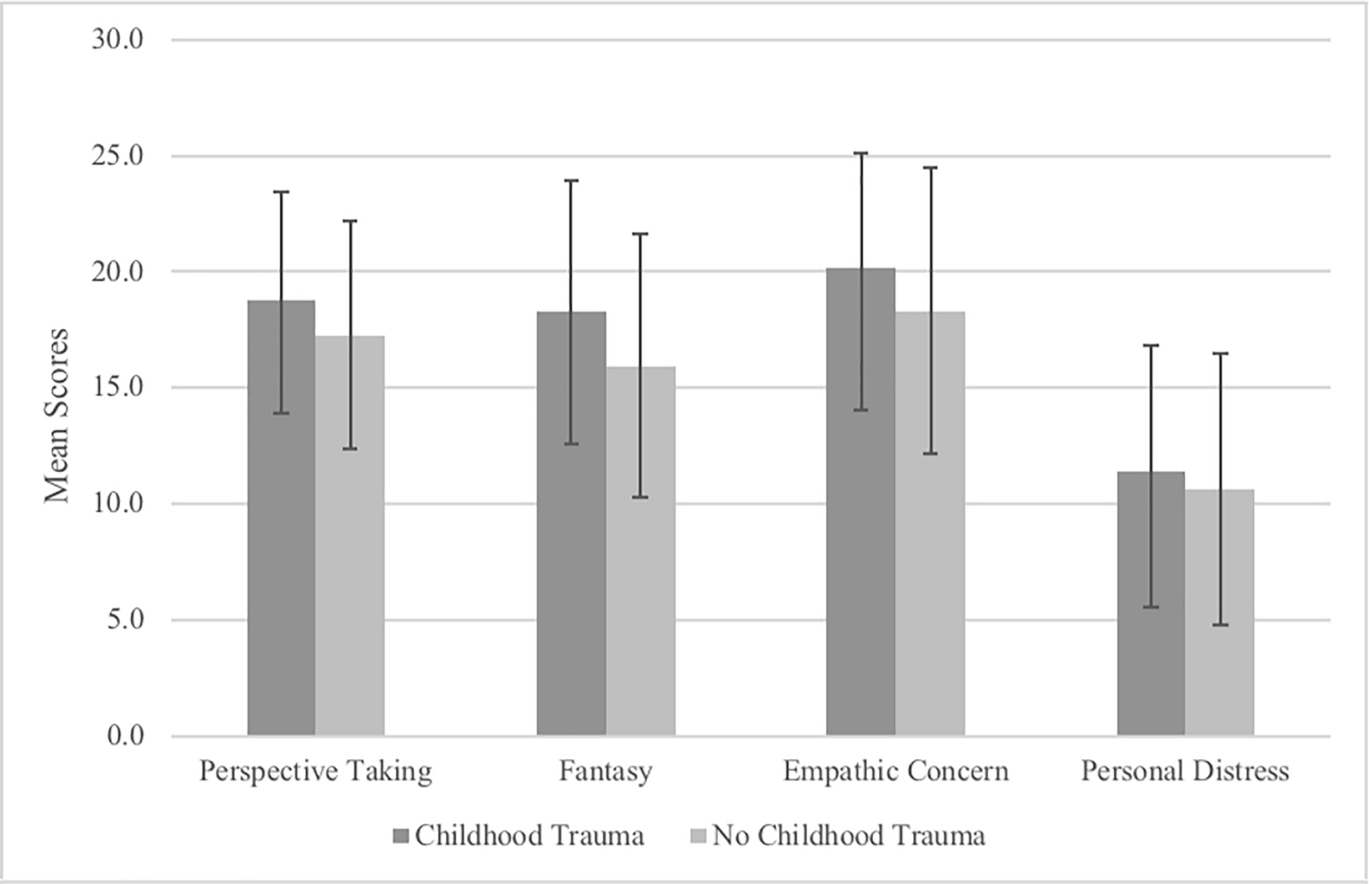
Differences in IRI facet scores by group type.

We performed ACOVAs with group type as the IV, Global empathy and facet scores as the DV, and sex as the covariate. The effect of childhood trauma on Global Empathy was significant (*F*(1, 438) = 11.84, *p* < .001, η_p_^2^ = .026). In terms of facet scores, there was a significant main effect of group type on Perspective Taking (*F*(1, 438) = 5.63, *p* = .02, η_p_^2^ = .013), Empathic Concern (*F*(1, 438) = 8.62, *p* = .003, η_p_^2^ = .019), and Fantasy (*F*(1, 438) = 5.42, *p* = .02, ηp2 = .012), with the trauma group scoring higher than the no trauma group on each. There was no main effect of group type on Personal Distress scores (*F*(1, 438) = 0.58, *p* = .45, η_p_^2^ = .001). We tested the assumption of homogeneity of regression slopes by entering interaction terms into the models, which yielded nonsignificant results, and therefore the assumption was tenable. Together, these results replicate and extend the findings from Study 1 by showing enhanced empathy scores in the trauma group compared to the no trauma group. Mean IRI facet scores are presented in Fig 2 for each of the groups (error bars are based on *SD*).

#### Trauma severity

We next examined zero-order correlations between the severity of the specified traumas and IRI scores. In general, the patterns of associations show that trauma severity was positively associated with IRI scores. Specifically, as can be seen in Table 2, Global scores were positively correlated significantly with the severity of experiencing a death and the severity of sexual abuse (*r*s = .24 and .25); Perspective Taking was positively correlated significantly with the severity of a death; and Empathic Concern was positively correlated significantly with severity of a death and severity of a parental upheaval (*r*s = .23 and .29).

**Table 2:** Correlations between IRI scores and severity of childhood traumas.

	Global Empathy	Perspective Taking	Empathic Concern	Fantasy	Personal Distress
**Death**	.24**	.17**	.29**	.11	.04
**Parental upheaval**	.14	.08	.23**	-.06	.11
**Sexual abuse**	.25*	.16	.18	.14	.14
**Violence**	.19	.08	.11	.12	.14
**Other**	.06	-.06	.02	.02	.15

*Note*: Cell entries are zero-order correlations between severity of childhood traumas and self-rated IRI scores. Ns = 238 (Death); 188 (Parental upheaval); 104 (Sexual abuse); 91 (Violence); and 120 (Other).

*p < .05

** p < .01.

#### Age during the trauma

We next examined the links between IRI scores and the age at the time of the trauma. ANOVAs revealed that there were no significant effects of age during the trauma on IRI scores (*F*s(5, 341) ranged from .14 for Global Empathy to 1.90 for Fantasy) and this result was further confirmed by post-hoc Tukey tests comparing each individual age group, with no significant differences between any of the groups.

## General discussion

### Summary of findings

Findings across multiple samples and measures showed that, on average, adults who reported experiencing a traumatic event in childhood had elevated empathy levels compared to adults who did not experience a traumatic event. Specifically, affective components of empathy were elevated across both samples and measures (i.e. the Affective Empathy facet of the EQ and Empathic Concern facet of the IRI). Cognitive components of empathy were elevated in Study 2 (based on scores on the Perspective Taking facet of the IRI), but only approached significance in Study 1 (based on scores on the Cognitive Empathy facet of the EQ). In general, the main effects observed were larger using the IRI than the EQ. Importantly, within the trauma groups, the severity the childhood trauma was positively linked to empathy levels. There was no effect on empathy in either Study 1 or 2 based on participant age at the time of the trauma.

There are three notable non-significant findings. First, there was no significant difference between groups for Cognitive Empathy in Study 1. This contrasts the results for the cognitive component of the IRI (i.e. Perspective Taking) which was found to be significantly different between the groups. This contrast may be due to the item construction of the EQ and IRI. In addition to items that refer to taking the perspective of another, the Perspective Taking component of the IRI also includes items referring to decision making (e.g. “I believe that there are two sides to every question and try to look at them both” and “I try to look at everybody's side of a disagreement before I make a decision”). And, in addition to items referring to ‘tuning’ into the thoughts and feelings of another, the Cognitive Empathy component of the EQ also contains items that refer to social situations (e.g. “I am quick to spot when someone in a group is feeling awkward or uncomfortable” and “I can sense if I am intruding, even if the other person doesn’t tell me”). These differences in scale construction may be responsible for the differences in the significance of the results. This warrants replication using behavioral measures of empathy, which is discussed below.

Second, there was no effect of trauma on the Social Skills component of the EQ. If higher scores on Social Skills were observed, we may have concluded that as a result of trauma, people may seek others for support, and as a therefore develop Social Skills that help to use social situations to meet their psychological needs. However, considering that no effect was found, this leads us to speculate that people may have developed and relied on internal resources, which may have contributed to their posttraumatic growth, and then not having to rely on social support for difficulties that arise in adulthood.

Third, there was no significant difference found for Personal Distress between the groups in Study 2. It would have been expected that the trauma group would have reported more distress as a result of the childhood trauma. However, it appears that the transition from childhood to adulthood and the process of posttraumatic growth may have alleviated feelings of personal distress. Therefore, empathy may be an ‘end-product’ of posttraumatic growth that is longer lasting than the initial personal distress that is expected to be felt immediately after a trauma. This is another indicator of adversity and resilience following childhood trauma.

### Limitations and future directions

#### Measurement

One of the main limitations of the present work is its reliance on self-report measurement. Critics might argue that those who have been traumatized may believe they have better empathy, perhaps as a consequence of frequently thinking about their own emotional state, and then self-report their empathy in an inaccurate way. Future research therefore needs to validate the heightened empathy found in the present study, perhaps by asking independent people who know the respondent to complete observer ratings. Concerns about confounds due to self-report bias could also be addressed by collecting behavioral data using performance tasks such as the “Reading the Mind in the Eyes” task [44], the “Faces Test” [45], or the Movie for the Assessment of Social Cognition (MASC) [46]. Further, the majority of participants in both studies reported a trauma during childhood. Future research could assess trauma with different instruments that are more qualitative in their group allocation than the methods used in the present studies.

The present research was also largely correlational, so although there was a link found between trauma and empathy, there may be psychological mechanisms fostering empathy that need to be identified. For example, the amount of social support following a traumatic experience may be crucial for the development of empathy. Further, psychological conditions and comorbidities that existed prior to the trauma and those that develop in response to the traumatic event may crucially impact the development of empathy after a trauma. Therefore, we were unable to examine if the observed elevated empathy developed as a feature of a clinical condition. Future research will need to design studies to test for transdiagnoses and the extent to which elevated empathy after a trauma may be accompanied by negative outcomes such as depression or positive outcomes such as resiliency. Further, the observed elevated empathy may be indicative of an increase of other constructs which empathy is closely related such as altruism. Future research will need to explore the role of empathy in comparison to other prosocial behaviors that may be observed after a trauma.

Biological and environmental factors may also lead an individual to have decreased empathy. Thus, there are likely varied pathways that lead toward or away from empathy after the experience of a trauma. These factors may be in part biological (e.g. neurological or genetic), social (e.g. family, community, or peer support), or individual (e.g. emotional regulation and copying strategies that are used). Future research could explore the development of empathy following a trauma using longitudinal methods. Further, because research suggests that empathy is in part linked to neurobiology [42], this line of research could also explore the links between trauma, brain development, and observed empathic behavior. Finally, both samples were predominantly White and therefore the generalizability of these findings to other ethnicities is limited and requires further testing, since empathy may be shaped differently across different cultures and people of varied socio-economic status. Finally, in both studies the number of individuals who reported a childhood trauma outnumbered the individuals who reported no childhood trauma. This disparity raises questions about the intersubjective experience of each participant and what they consider a traumatic event. This is particularly relevant for individuals who selected the “other” option in the trauma item. This is also raises more theoretical and philosophical questions about the extent to which trauma is perceived to be “normal” or “abnormal” by both science and the general public.

#### Clinical implications

The finding of elevated empathy levels in adults with childhood trauma might at first glance appear to contradict the accumulating evidence of reduced mentalizing capacity in individuals who have experienced trauma [47,48]. We would suggest, however, that the findings of elevated empathy in the non-clinical populations are congruent with the theory in ways that have important clinical implications [49]. There is some evidence that a stronger capacity for mentalizing is protective and that the ability to mentalize a trauma could be a resilience factor. Early findings in this area [50] have been replicated by Berthelot et al. [51], who found that mothers who had high mentalizing in relation to their traumatic experience had fewer children with disorganized attachment (37%) than mothers with low mentalizing (67%). Such findings [52,53] could be interpreted as suggesting that mentalizing contributes to a process of resilience that is also reflected by the findings here. The fact that the sample in this study was taken from non-clinical population leaves us with the intriguing possibility that the elevated empathy measured (if taken as a marker for higher mentalizing) has helped to protect the individuals concerned from some of the negative mental health outcomes associated with experiencing adversity in childhood.

However, the extent to which these findings extend directly to clinical populations (in which the concept of mentalizing may be more relevant) is not straightforward. We would caution against the simple equation of elevated empathy with strong mentalizing capacity. Empathy and mentalizing are related but different constructs. Even though mentalizing has affective components [54], it is often equated with cognitive empathy (also called ‘theory of mind’). Empathy is a separate concept because it also encompasses the ability to understand another’s mental state (cognitive empathy) and to react with an appropriate emotion to the mental states of others (affective empathy). Another key difference is that mentalizing is the ability to understand the mental states of both others and the self [55] and the mentalizing model assumes a virtous cycle of increasing complexity linking the two (viz. I learn about myself through others’ reactions to me, which I understand increasingly well through improved self understanding that permits me to place myself increasingly effectively into their shoes) [55].

An example of how clinically the relationship between empathy and symptoms are complex is illustrated in BPD. Undue weight on affective empathy might manifest clinically as maladaptively high levels of sensitivity, leading to vulnerability to emotional contagion, a weaker sense of self, and a lack of capacity for personal affect regulation (characteristics typically found in individuals with BPD) [56]. Such characteristics underpin what has been described as the borderline ‘empathy paradox’ [57]. For example, individuals with BPD have been found to have heightened sensitivity to others’ emotional cues [58], and show superiority in attributing mental states on the basis of emotional cues [59]. However, this weighting towards affective empathy is often met with a deficit in the area of cognitive empathy in individuals with BPD [60]. In contrast to the mentalizing profile associated with BPD, an imbalance towards cognitive empathy might manifest as a strong capacity to read and understands others’ mental states, but not to resonate with them on an emotional level; this might be associated most typically with individuals with ASPD. Recent research on hyper-mentalizing in adolescents with emerging BPD (who also tend to have trauma histories) indicates that these individuals manifest increased rather than decreased mentalizing in relation to the Movie Assessment of Social Cognition [61,62]. This test differentiates an apparent concern with the mental states of protagonists from a genuine accurate concern [46]; the measures of empathy used in this study do not make that distinction, and this may be a useful future direction for research. Taken together, although the findings from the present study suggest that elevated empathy after a trauma may play a protective role in non-clinical samples, however, this phenomenon may have a more complex picture in more clinical samples and needs to be investigated in future research.

## Conclusions

The present studies show that as a result of the traumatic experience, people are able to better understand the emotional and mental states of others. This interesting finding adds to emerging theory and research that shows that adversity can lead to posttraumatic growth, but it needs to be validated using measures other than self-report and extended to clinical populations. Future research should explore how a person’s increased empathy following a traumatic event may lead them to actually taking action to help others who are experiencing suffering similar to that which they experienced.
